# Full Chromosomal Relationships Between Populations and the Origin of Humans

**DOI:** 10.3389/fgene.2021.828805

**Published:** 2022-02-02

**Authors:** Rui Dong, Shaojun Pei, Mengcen Guan, Shek-Chung Yau, Changchuan Yin, Rong L. He, Stephen S.-T. Yau

**Affiliations:** ^1^ Yau Mathematical Sciences Center, Tsinghua University, Beijing, China; ^2^ Yanqi Lake Beijing Institute of Mathematical Sciences and Applications, Beijing, China; ^3^ Department of Mathematical Sciences, Tsinghua University, Beijing, China; ^4^ Information Technology Services Center, The Hong Kong University of Science and Technology, Kowloon, Hong Kong, China; ^5^ Department of Mathematics, Statistics and Computer Science, University of Illinois at Chicago, Chicago, IL, United States; ^6^ Department of Biological Sciences, Chicago State University, Chicago, IL, United States

**Keywords:** 1000 genomes project, reconstructed sequences, natural vector, divided natural vector, population genetics

## Abstract

A comprehensive description of human genomes is essential for understanding human evolution and relationships between modern populations. However, most published literature focuses on local alignment comparison of several genes rather than the complete evolutionary record of individual genomes. Combining with data from the 1,000 Genomes Project, we successfully reconstructed 2,504 individual genomes and propose Divided Natural Vector method to analyze the distribution of nucleotides in the genomes. Comparisons based on autosomes, sex chromosomes and mitochondrial genomes reveal the genetic relationships between populations, and different inheritance pattern leads to different phylogenetic results. Results based on mitochondrial genomes confirm the “out-of-Africa” hypothesis and assert that humans, at least females, most likely originated in eastern Africa. The reconstructed genomes are stored on our server and can be further used for any genome-scale analysis of humans (http://yaulab.math.tsinghua.edu.cn/2022_1000genomesprojectdata/). This project provides the complete genomes of thousands of individuals and lays the groundwork for genome-level analyses of the genetic relationships between populations and the origin of humans.

## Introduction

Genetic analysis of hominins has yielded insights about modern populations, superpopulations and their histories. Meanwhile, the relationship between modern and ancient humans remains controversial and of great importance for understanding our phylogenetic position in the tree of life. As the largest public catalog of human variation and genotypes, the 1,000 Genomes Project, initiated by the International Genome Sample Resource (IGSR), aims to discover genotypes and provides accurate haplotype information for all forms of human DNA polymorphism based on 2,504 individuals from 26 populations all over the world (The 1000 Genomes Project Consortium, 2010). Besides the regular study about the relationships between genotype and phenotypes such as diseases, this provides an ideal material for phylogenetic analysis about relationships among populations on genome level. On the subject of human origin, no unanimous conclusion has been reached so far due to the conflict results mainly caused by different choices of genes, incomplete data and different data types (Wickett et al., 2014). Though most researchers acknowledge the “out-of-Africa” assumption, most studies are performed based on the neutral theory of molecular evolution, whose theoretical proof hasn’t not rarely examined (Leffler et al., 2012). Counterexamples of neutral theory include genome-wide constraints such as fold pressure and GC-pressure, mtDNA and nuclear genome compatibility (Huang, 2016). The neutral theory, which largely sounds as a null model and framework of pre-saturation evolutionary processes, has met with great difficulty as an explanatory framework for most molecular evolutionary phenomena and as such should not have been so freely used to account for genetic diversity patterns (Yuan et al., 2019). Thus, the “out-of-Africa” assumption requires a reasonable model and should be conducted on full chromosome data rather than a few genes or local region. The relationships between populations should be inferred at the genomic level, which reflects a complete view of individuals in a population. A comprehensive description of human evolutionary patterns is essential for studying the relationships between modern populations and, if combined with the information on ancient hominins, can be used to study the origin of modern humans.

On the other hand, fossil records of ancient hominins have been found recently and are the most direct material with which to study human origins in archeology. Sequencing results for such fossils allow us to analyze them at the genome level. The Neandertals were recognized as a distinct group of hominids with numerous fossils as well as stone tool assemblages (Krings et al., 1997; Heyes et al., 2016; Douka et al., 2019). Nevertheless, few fossils of the Denisovans, another distinct member of the *Homo* genus, have been found. Analysis of the DNA from the Neandertal and Denisovan fossils has great potential to provide insight into their population histories and relationships with modern humans, but progress has been limited due to the rarity of samples and damaged state of the DNA (Kalvin et al., 1995). Although complete genomes of ancient hominins are extremely difficult to obtain, mitochondrial genomes have already been sequenced by primer extension capture (PEC) and other techniques (Briggs et al., 2009). However, most research on ancient mitochondrial genomes focuses on local gene information instead of the global feature of the genome, and suffers from the dispute of neutral theory.

Besides the traditional alignment-based methods which requires the neutral theory assumption, many alignment-free methods have been proposed in recent years, such as feature frequency profiles (FFP) (Jun et al., 2009), power spectrum method (Yin et al., 2014; Dong et al., 2018; Pei et al., 2019), the Natural Vector (NV) method and its extensions (Deng et al., 2011; Wen et al., 2014; Dong et al., 2019) with successful applications (Huang et al., 2014; Zheng et al., 2015; Dong et al., 2017). In contrast to alignment-based methods, NV doesn’t rely on human intervention or arguable assumptions such as neutral theory, and the theoretical proof of NV is solid as fully described in (Deng et al., 2011). In this project, we propose an improved version of NV method named Divided Natural Vector (DNV), which integrates local nucleotide information together with global distribution data. A parameter, k, predetermined by the algorithm, indicates how many segments the original sequence should be divided into. With an appropriate value of k, the divided natural vector method achieves the same or better results compared with those from alignment-based methods, using less computation time, as shown in Supplementary Figure S1. Another advantage of both NV and DNV is high time-efficiency and the compatibility of mathematical techniques derived from the conversion from biological sequences to mathematical vectors. This allows a simple but effective idea of taking average to extract features on populational level without variability from personal genomes, but difficult to conduct on DNA sequences directly. As illustrated in *Materials and Methods* and Supplementary Figure S2, the first step of our project is to reconstruct 2,504 individual genomes from variant calling results of the 1,000 Genomes Project and reference genomes. Then we applied DNV on all reconstructed genome sequences and extract the feature for each population, to measure the genetic similarity among populations. We also performed the DNV approach on a larger dataset including mitochondrial genomes from the 1,000 Genomes Project, Human Mitochondrial database, L0 mitogenomes and sequencing results from fossils of ancient humans, to gain insight into our origin. The workflow of this project is summarized in Supplementary Figure S3.

## Results

### Total Autosome Nucleotide Tree

**FIGURE 1:**
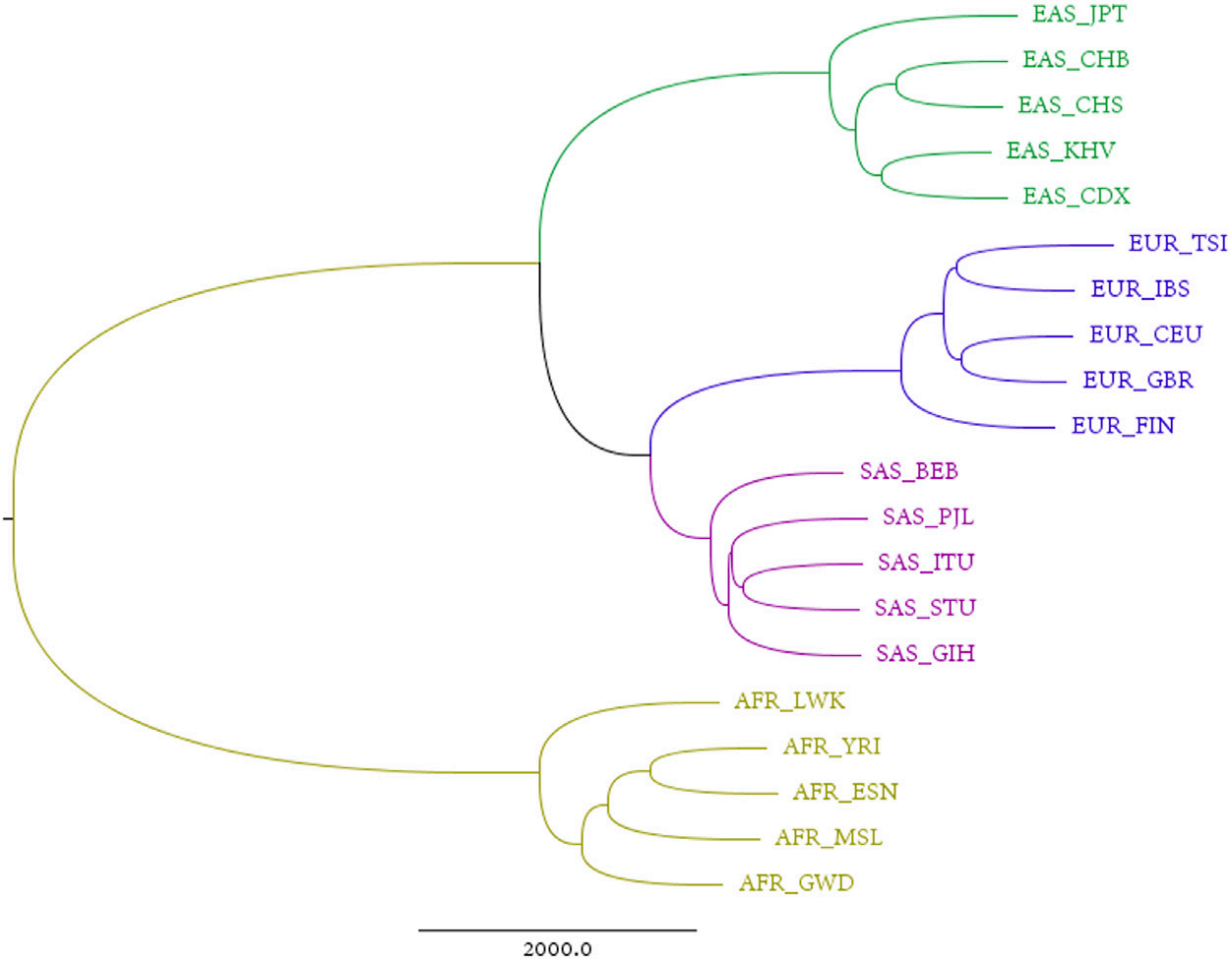
Total autosome nucleotide tree (TANT) of 20 non-American populations based on the DNV (k = 4) approach.

An alignment-free approach, the divided natural vector approach, is proposed in Supplementary Material. We applied this approach to the reconstructed sequences. Each individual has a reconstructed genome, consisting of 22 pairs of autosomes, one pair of sex chromosomes (XY for male, and XX for female), and a mitochondrial genome. Considering the maternal inheritance of mitochondria, that sex chromosomes differ between males and females, and that autosomes comprise over 90% of the human genome, we reconstructed phylogenetic trees by integrating all autosomes. The sum of distance matrices of 22 pairs of autosomes reflects the dissimilarities between populations derived from all autosomes and is an ideal metric for phylogenetic analysis. The corresponding tree generated from this matric is therefore called the “total autosome nucleotide tree” (TANT). Among the 26 populations from the 1,000 Genomes Project, the six current American populations are actually combinations of humans from all over the world, which introduces noise into studies on the origin of humans. The phylogenetic tree in Figure 1 shows that all the other four superpopulations, i.e., African, European, East Asian, and South Asian, are monophyletic groups. This contrasts with the model of sequence evolution, in which only non-African individuals are monophyletic (Briggs et al., 2009). Besides, we also constructed phylogenetic trees using UPGMA/Neighbor-Joining and DNV/NV with results shown in Supplementary Figures S4, S5. Results based on single chromosomes can be found in Supplementary Figures S6–S8, and we also calculated the Robinson-Foulds distances for chromosome X and each autosome in Supplementary Table S1.

### Natural Graph

**FIGURE 2:**
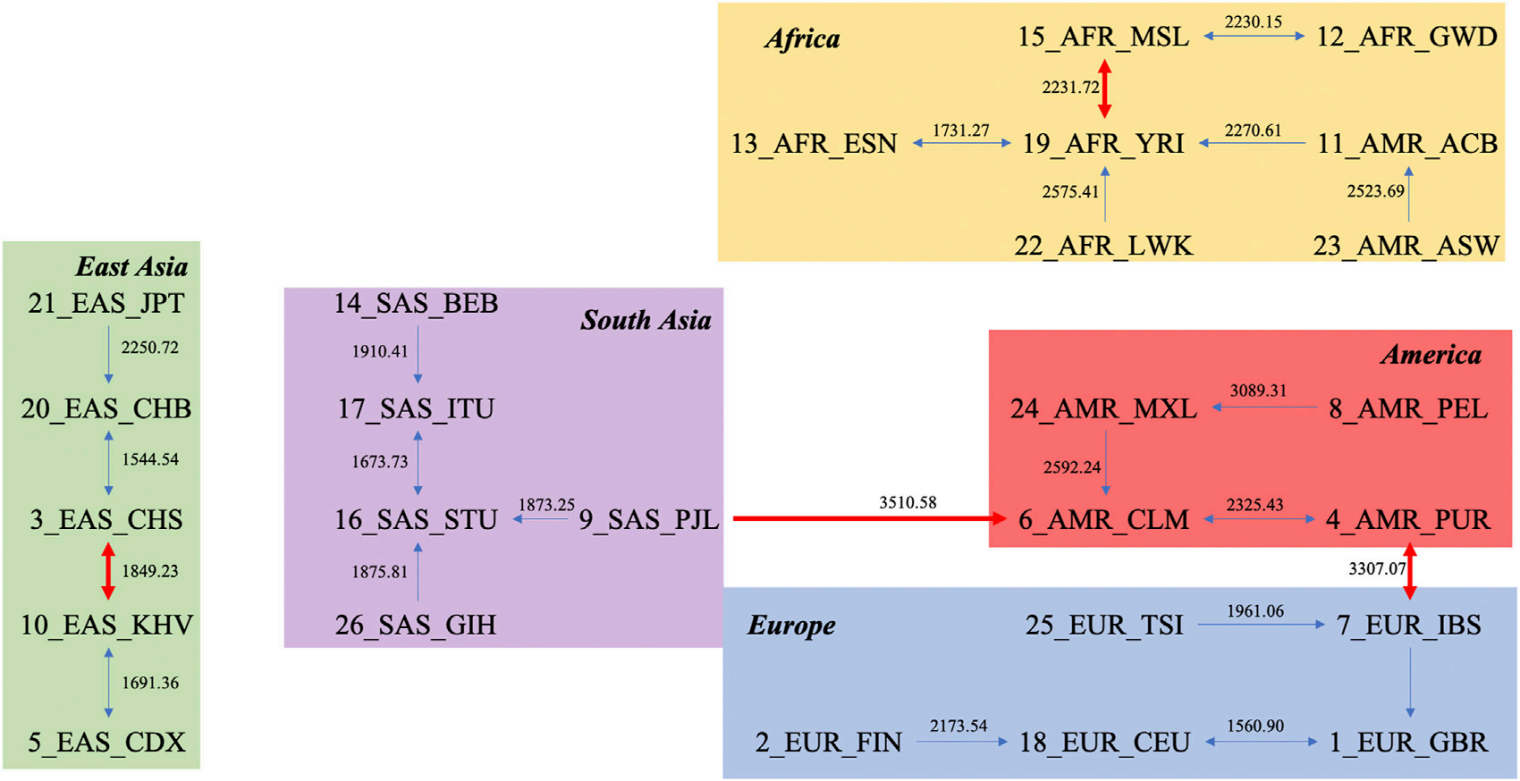
Natural graph based on the distance matrix obtained by the DNV method.

Another novel way to study the relationship between units is *via* the natural graph approach. By finding every unit’s closest neighbor based on layers, the relationships between populations can be intuitively observed. The natural graph for the DNV approach is presented in Figure 2. The first layer, which classifies each population with its closest neighbor, is shown as thin blue lines, and the second layer, which reflects the relationships between groups generated from the first layer, is shown as thick red lines. Figure 2 shows that Americans are closely connected to other superpopulations, while African populations are more distinct from other populations (largest average distance between Africans and other monophyletic superpopulations). The distance within each subpopulation is presented in Supplementary Table S2, also supporting the “out of Africa” hypothesis, given by the fact that larger genetic distances reflect higher diversity in genomes, and correspondingly, larger division among Africans compared to populations on other continents.

### mtDNA Results Based on 1,000 Genomes Project Data

**FIGURE 3:**
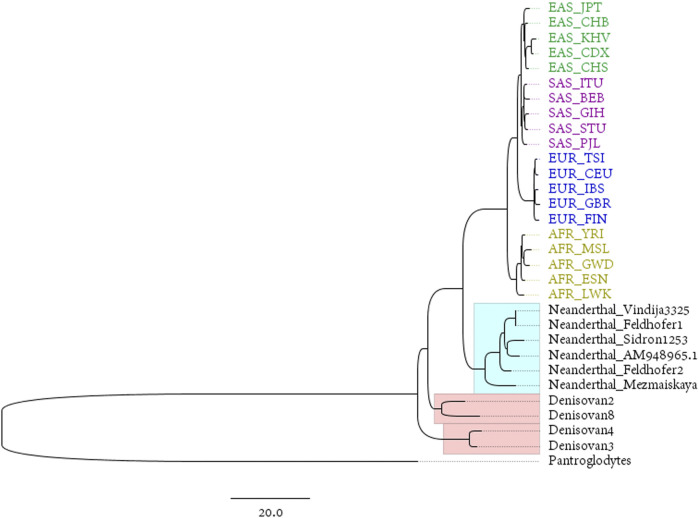
BIONJ tree based on the distance matrix obtained with the DNV method from the mitochondrial data of 20 modern populations from 1,000 Genomes Project and 10 ancient hominins (with Pan troglodytes as the outgroup).

The Neanderthals and the Denisovans are two extinct species or subspecies of hominins in the genus Homo. Mitochondrial DNA was extracted from fossils of both taxa by PEC (Briggs et al., 2009) or other techniques. This provides an ideal material for studying the maternal inheritance patterns of ancient and modern humans. The reconstructed mitogenomes from the 1,000 Genomes Project represent modern populations in the dataset, while the complete mitogenomes of 4 Denisovans and 6 Neanderthals represent ancient hominins (the mitochondrial genome of Pan troglodytes is used as an outgroup to root the phylogenetic tree). The phylogenetic tree is shown in Figure 3.

The Luhya in Webuye, Kenya (LWK), population is the closest modern population to the root of the tree in Figure 3. This is the only population in the 1,000 Genomes dataset that are widely distributed in eastern Africa. Some of the earliest hominin skeletal remains have been found in this region, including those discovered in the Awash Valley of Ethiopia, as well as the Koobi Fora in Kenya. “Lucy”, the well-known skeleton of a female from the hominin species Australopithecus afarensis, was discovered in 1974 in the Awash Valley and dated to approximately 3.2 million years ago (Ma). According to the predominantly held belief among most archeologists, East Africa is the area where anatomically modern humans, at least females, first appeared.

### Inheritance Pattern and Relationships Among Super-Populations

In Figure 1, European populations show a closer relationship to South Asian, than to East Asian populations on the analysis of TANT which contains the information of all autosomes. It is easy to observe the same structure in Chromosome X for all females shown in Supplementary Figure S7A. This coincidence can be explained by the same parental inheritance for autosomes for all individuals and Chromosome X for females, i.e., offspring inherits one haploid from each parent.

For paternal inheritance of Chromosome Y of all males, Supplementary Figure S7B suggests that Europeans are more similar to East Asian compared to South Asian. Frequent wars between Europeans and East Asians in human history explain the reasons for this phenomenon as wars involves mainly men. Besides, in Supplementary Figure S7B, Finland lies in the branch of East Asia and the long history connection between Finland and Russia since 1809 may contribute to this result.

For maternal inheritance, mitochondrial genomes lead to a conclusion different from either TANT or Chromosome Y. In Figure 3 and Supplementary Figure S8, East Asians and South Asians share more genetics in comparison with Europeans. Geographical factors such as flat terrain, and similar language structures may contribute to the frequent communications within Asians, meanwhile Ural Mountains and Ural River separates Eurasia into two continents. On the contrary of paternal inheritance, wars play little role in the maternal inheritance and females are relatively conservative and subject to geography and linguistics due to historical reasons.

Therefore, parental, paternal and maternal inheritance patterns lead to different phylogenetic trees using the same DNV method in our work, and for more details and explanation on the results, please see Supplementary Material Section 1.1.3.

### Human Origin Based on the Human Mitochondrial Database

**FIGURE 4:**
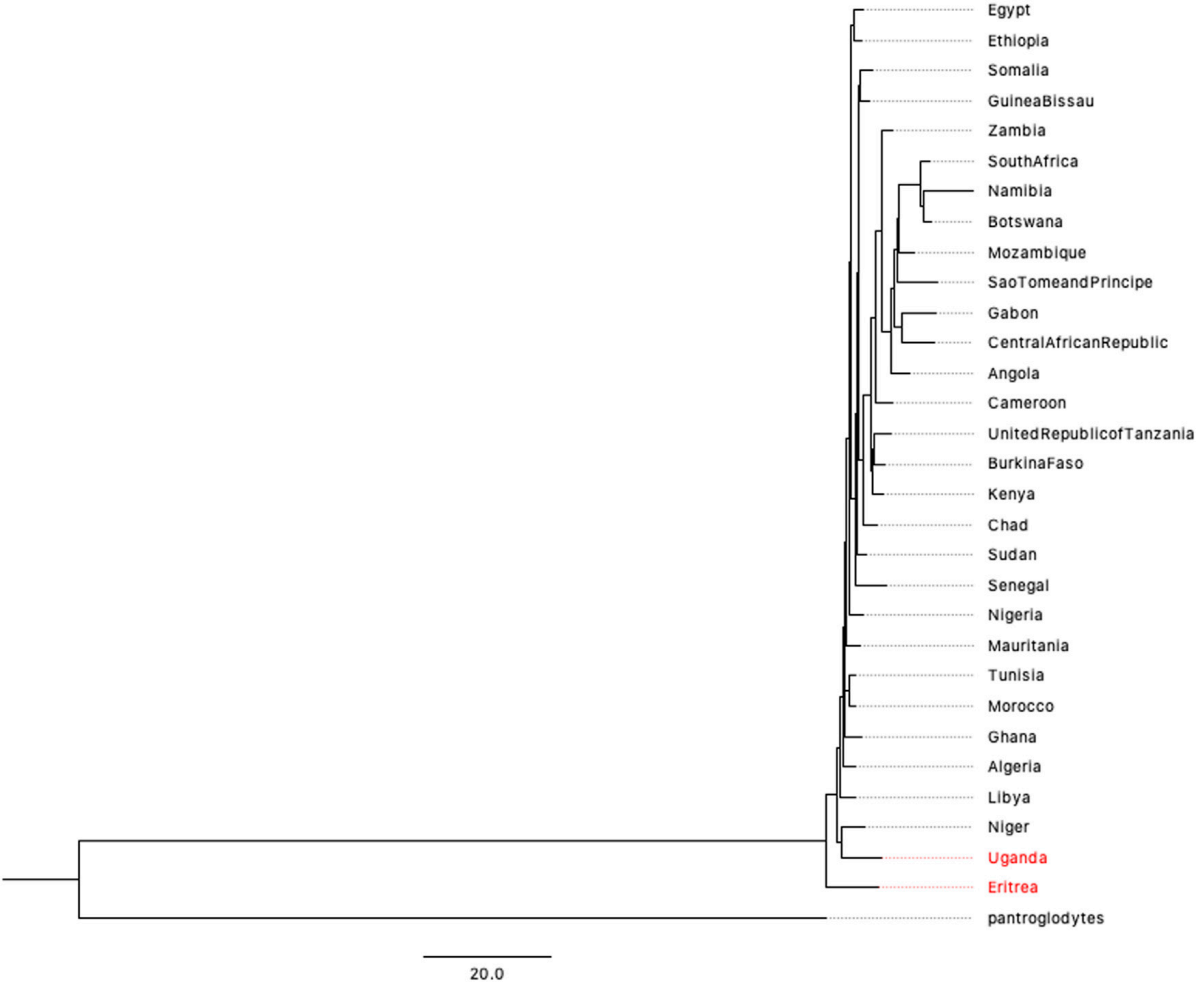
BIONJ tree of 30 countries on African continent based on the mitochondrial data of 2932 individuals (with Pan troglodytes as the outgroup).

Although the 1,000 Genomes Project covers most populations in the world, the five African populations are concentrated on the eastern and western coasts of Africa. The accurate prediction of human origin requires a dataset that includes most countries on the African continent. After filtering data from the Human Mitochondrial database (HmtDB) (https://www.hmtdb.uniba.it) (for more details, please see Supplementary Material), we analyzed 2932 high-quality mitochondrial genome sequences from 30 countries on African continent. The phylogenetic tree is shown in Figure 4. Eritrea lies on the branch closest to the root of the tree. A previous study by Italian researchers showed a possible link between *Homo erectus* and *Homo sapiens* (Macchiarelli et al., 2004; Zanolli et al., 2014; Medin et al., 2015). The link is related to one of the oldest hominids, named “Madam Buya” in Eritrea. Along with an adult cranium (UA 31), which displays a blend of *Homo erectus*-like and derived morpho-architectural features, and three pelvic remains, two isolated permanent incisors (UA 222 and UA 369) have also been recovered from the Homo-bearing outcrop of Uadi Aalad dated to 1 Ma. During the last interglacial period, the Red Sea coast of Eritrea was occupied by early anatomically modern humans. Eritrea also has a long and close affinity with Ethiopia, where a variety of fossils have been discovered. The second closest branch is the population of Uganda, another country in eastern Africa. The phylogenetic trees presented in Figures 3, 4 provide genetic evidence of the origin of humans, which is inferred to be in eastern Africa.

### Phylogenetic Analysis Focusing on L0 Mitogenomes

Recent research (Chan et al., 2019) claimed that human origins in a southern African palaeo-wetland, and we explored the dataset they used in the literature as well. Two datasets, containing 6,334 and 1217 L0 mitogenomes respectively, were both firstly filtered under the same condition as illustrated in Supplementary Material. After filtration, we have 1733 and 563 sequences in two sets respectively. Clustal Omega algorithm and FASTME were used to construct the phylogenetic tree, and both trees suggest that an individual with accession number KF672800.1 is the deepest branch in the tree. This sample is from Kenya, therefore supports our conclusion that human, at least females, originated from eastern Africa.

In (Chan et al., 2019), there are very few mitogenomes from eastern Africa compared to the mitogenomes form southern Africa. Therefore, we also generate another set covering both the qualified sequences published in the literature and additional sequences from eastern Africa to achieve balance. This new set contains 3,495 individuals and the result also suggests the eastern Africa as the origin of human based on mitochondrial genomes. A thorough study was performed on the two specific sequences that are close to the root of the phylogenetic tree (EU092699.1 from Mozambique and EU092943.1 from Ethiopia), and we compare the similarity of these two sequences to mtDNA of gorilla (KM242275.1) using NCBI BLAST method. The identity between Ethiopia and gorilla is 13944/15584, higher than between Mozambique and gorilla 13939/15584. Besides, the third to sixth sequences closest to the root are all sequenced from samples in eastern Africa (Chad, Kenya, Ethiopia and Ethiopia). Therefore, based on the evidence from mitochondrial genomes, the origin of human should lie in eastern Africa rather than southern Africa.

The tree files can all be found on GitHub (https://github.com/YaulabTsinghua/Human-Origin-1kGP).

## Discussion

This project proposes the DNV approach and applies it on the data from the 1,000 Genomes Project, providing insight into the genetic relationships among the main human populations worldwide. Alignment-free methods have largely been ignored as a powerful tool for studying the human genomes and population relationships, and our research fills this gap. With the successful application of DNV described in *Natural Vector and Divided Natural Vector* and Supplementary Material, we prove that DNV outperforms NV on the test set and enjoys higher time-efficiency compared to alignment methods. DNV offers greater speed as recorded in Supplementary Table S3, which is significant advantage over alignment-based approaches, especially dealing with genomes data.

The relationship among populations is studied under different inheritance patterns based on whole-genome data of 2,504 individuals, and the results show that parental, paternal and maternal inheritances lead to different evolutionary results in the corresponding phylogenetic trees. Similarity between East Asians and Europeans can be explained by the frequent wars in history, mainly conducted and participated by males. Asian females share higher genetics within Asia compared to them with European females, reasons of which includes geographical and linguistics factors. Autosomes and Chromosome X for females, however, generate a result different from both, but can be viewed as a balanced average of the paternal and maternal results.

As to the topic of origin of human, the large average distance within African populations suggests a high diversity on genome level, and phylogenetic analysis approves the origin of Africa as well. This is the solid evidence for the ‘out-of-Africa’ model because the distance is calculated based on all autosomes instead of a local region or gene. To explore more details about the location of origin, we collect external data source from other mitochondrial databases to cover the whole African continent, and compared them all with the ancient mitogenomes extracted from fossils. Results in Figures 3, 4 both suggest that eastern African is more likely to be the origin of human and Eritrea is with the highest possibility. Results based on the 1,000 Genomes Project suggests that Kenya population is the closest which also supports the conclusion of eastern Africa, though the data source covers only five populations in Africa. The assertion depends on the appropriate outgroup, in this project, chosen as the mitogenome of pan troglodytes. However, due to the inconsistency of number of autosomes of human and other primates, root of phylogenetic tree for autosomes is difficult to identify based on our method. Research results based on mitochondrial genomes reflect the maternal inheritance, and if we consider parental as a mixture of paternal and maternal, plus the fact that early migrations are less frequent than nowadays, the conclusion of eastern Africa origin still stands. Our project provides a satisfying answer to the debate generated by the use of different datasets and analytical methods. Resolving such phylogenetic relationships is important for comparative genomics, which may be further applied to the study of human diseases. Most studies published to date have applied alignment algorithms to local variants, which neglects the key information hidden in whole-genome data.

In summary, the proposed DNV method resolves the issue of alignment methods caused by insufficient data and our whole-genome data source improves phylogenetic reconstructions using the complete evolutionary record within each individual’s genome (Javis et al., 2015). The reconstructed genomes are stored on our server (http://yaulab.math.tsinghua.edu.cn/2022_1000genomesprojectdata/) and can be further used for any genome-scale analysis of humans. The project lays the groundwork for genome-level analyses of the genetic relationships between populations and the origin of humans.

## Materials and Methods

### Reconstruct the Genomes Data From VCF File

The 1,000 Genomes Project performs alignment using the Burrows-Wheeler transformation (BWA) mapping algorithms for low coverage sequencing in phase 3 of the whole project. Variant calls are always released in variant call format (VCF) (Li and Durbin 2009; Danecek et al., 2011). Based on the reference sequence and the VCF record data, it is possible to reconstruct the sequences for each individual, by replacing the nucleotides on the reference sequence with the variants at the corresponding positions. Although the current coverage of sequencing technology is insufficient to really ‘reconstruct’ the genome of each individual, it is still a promising approach for studying the relationships among populations, by using averaging to decrease the noises in the reconstructed dataset.

BCFtools is a set of utilities that manipulate variant calls in the VCF and its binary counterpart BCF. The command ‘consensus’ in the set of BCFtools can create a consensus sequence by applying VCF variants to a reference fasta file (Danecek and McCarthy, 2017). An example of a VCF file with five individuals is shown in Supplementary Figure S1A. The first individual, named as “HG00096”, has no variants at these positions, therefore, its corresponding sequence remains the same as the reference sequence. The second individual “HG0770” has a variant of “C” at POS = 10, while the reference nucleotide is a “T”. Therefore, we change the 10th nucleotide on the reference sequence from “T” to “C”, so as the 26th nucleotide from “C” to “T”. Any positive integer in the main data means the corresponding alternative in the ALT column. For “HG01992” and “HG02230”, they both have the second alternatives on position 55, then we locate the 55th nucleotide, and because there is more than one nucleotide in the REF column, the corresponding segment to be replaced has more than one nucleotide. “POS = 55” refers to the first nucleotide in this segment, and the second alternative, “CATTTT”, is used to fill in the segment in this window. If we only consider the data in Supplementary Figure S1A, individuals “HG00096” and “HG02231” should have the same reconstructed sequences as the reference sequence, and “HG01992” and “HG02230” should share the same reconstructed sequences. The corresponding reconstructed sequences are shown in Supplementary Figure S1B.

In the 1,000 Genomes Project, both short variations and structural variations are detected. Most of the complicated variations consist of copy number variations (CNV). CNV is a phenomenon in which sections of the genome are repeated and the number of repeats in the genome varies between individuals in the human population. It often becomes unclear whether or not a region is an overlap or a duplicated region. Usually, the POS coordinate is based on the leftmost possible position of the variant, which indicates that the accuracy of estimation could strongly affect the duplicated region. Therefore, in our study, the structural variants are not taken into consideration, and could be implemented into the current results in further analysis.

### Humans Diploidy and Pairs of Chromosomes

Besides mitochondria genomes, the human genome includes the 23 chromosome pairs, 22 of which are called autosomes, with the other pair commonly known as the “sex chromosome”. The diploidy of human individuals results in the possibility of different sequencing results on the same chromosome pair and it is impossible to detect if a chromosome is patrilineal or matrilineal with current sequencing techniques. For an individual, we picked one chromosome of each pair to reconstruct two separate chromosome sequences, named “a” and “b”, one from the father and the other from the mother. Thus, regarding autosomes, every individual has Chr1a, Chr1b; Chr2a, Chr2b; …; Chr22a, Chr22b in the reconstructed autosomes. Please note that it is possible that “a” means matrilineal in the Chromosome 1, but means patrilineal in the Chromosome 2.

For the 23rd chromosome pair, i.e., the sex chromosome pair, females have a pair of X chromosomes, and males have one X chromosome and one Y chromosome. Using female data to study the phylogeny of the X chromosome, and male data to study Y chromosome, makes it possible to separate any possible noise in the pseudo-autosomal region, although the number of samples are decreased by half. Pseudo-autosomal regions are homologous sequences of nucleotides on the X and Y chromosomes, but on different positions, partly due to their markedly different scales. Therefore, the existence of pseudo-autosomal regions increases the difficulty of studying the phylogenetic relationships based on gender. In the situation of female data, procedures are identical to the autosome cases, while for male data, it degenerates to a simpler case: one sequence (of a Y chromosome) represents a male individual.

### Natural Vector and Divided Natural Vector

For each chromosome (except the Y chromosome), each individual’s pairs of chromosomes are reconstructed. Then we applied the alignment-free natural vector method to this dataset.

The natural vector method was first proposed by Yau and his team (Deng et al., 2011). The natural vector of a sequence encodes the distribution of four nucleotides. Yau has proven mathematically that there is a one-to-one mapping between a sequence and its natural vector (Deng et al., 2011). Given any nucleotide sequence, the natural vector method maps it into a vector/point in real Euclidean space, where for each type of nucleotide, the first parameter describes its quantities in the sequence, the second gives the mean values of total distances to the first nucleotide in the sequence, and the last consists of the normalized central moments. The normalized central moments are defined as follows:

$${\text{D}}_{\text{j}}^{\text{k}}=\overset{{\text{n}}_{\text{k}}}{\underset{\text{i}=1}{\sum }}\frac{(\text{s}\left[\text{k}\right]\left[\text{i}\right]-{\text{μ}}_{\text{k}}{)}^{\text{j}}}{{\text{n}}_{\text{k}}^{\text{j}-1}{\text{n}}^{\text{j}-1}},\text{j}=2,\ldots ,{\text{n}}_{\text{k}}$$

where k = A, C, G, T. Here, ${\text{n}}_{\text{k}}$ denotes the number of nucleotide k in the DNA sequence and n is the length of the sequence. s[k][i] is the distance from the first nucleotide (regarded as origin) to the *i*th nucleotide k in the sequence. ${\text{T}}_{\text{k}}=\overset{{\text{n}}_{\text{k}}}{\underset{\text{i}=1}{\sum }}\text{s}\left[\text{k}\right]\left[\text{i}\right]$ denotes the total distance for each set of A, C, G, T from the origin, k = A, C, G, T. ${\text{μ}}_{\text{k}}=\frac{{\text{T}}_{\text{k}}}{{\text{n}}_{\text{k}}}$ is the mean value of the distances of nucleotide k. The central moments can be written as < ${\text{D}}_{2}^{\text{A}},{\text{D}}_{2}^{\text{C}},{\text{D}}_{2}^{\text{G}},{\text{D}}_{2}^{\text{T}},\ldots ,{\text{D}}_{{\text{n}}_{\text{A}}}^{\text{A}},{\text{D}}_{{\text{n}}_{\text{C}}}^{\text{C}},{\text{D}}_{{\text{n}}_{\text{G}}}^{\text{G}},{\text{D}}_{{\text{n}}_{\text{T}}}^{\text{T}}$ >. It is obvious that higher moments converge to 0. In common practice, the first 12 dimensions of the natural vector: < $n_{A},n_{C},n_{G},n_{T},\mu_{A},\mu_{C},\mu_{G},\mu_{T},{\text{D}}_{2}^{A},{\text{D}}_{2}^{C},{\text{D}}_{2}^{G},{\text{D}}_{2}^{T}$ > are used, and have been found to perform well. Once their natural vectors have been calculated, the similarity between sequences can be measured by the Euclidean distance between the corresponding vectors.

Consequently, each pair of chromosomes, i.e., sequences, can be converted to a pair of natural vectors, and then those natural vectors can be used for further analysis. Based on the uncertainty of which one in the pair is patrilineal, they should be assigned equal weight in the study. In mathematics, one of the best ways to describe a pair of vectors is to take their average position. Consider two points in n-dimensional space, $\left(x_{1},\;x_{2},..,x_{n}\right)$ and $\left(y_{1},\;y_{2},..,y_{n}\right)$ , their average position is $\left(\frac{x_{1}+y_{1}}{2},\frac{x_{2}+y_{2}}{2},\ldots ,\frac{x_{n}+y_{n}}{2}\right)$ . In the case of the traditional natural vector method, n = 12. Thus, we can use an individual’s 22 autosomes to get 22 average personal natural vectors. For a female individual, extra average natural vector can be generated from her pair of Chromosome X and the Robinson-Foulds distance between BIONJ trees from chromosome X to each autosome is shown in Supplementary Table S2.

In the final phase of 1,000 Genomes Project, data from 2,504 individuals was made available to the worldwide scientific community through freely accessible public databases. Therefore, regarding Chromosome 1, we have 2,504 pairs of Chromosome 1, corresponding to 2,504 individuals. The 2,504 pairs of chromosomes are represented by 2,504 pairs of natural vectors, also by 2,504 average personal natural vectors, which reveal mathematically the hidden information in the original reconstructed sequence, i.e., from reference sequence and the SNP (single nucleotide polymorphism) and other variants.

Each population contains about 100 individuals in the dataset, for example, “ACB” is short for “African Caribbean in Barbados”, which consists of 96 individuals, corresponding to 96 average personal natural vectors of Chromosome 1. The idea of taking an average is applied again here to find the representative point of each population, fixed one chromosome, which is the center of all the average personal natural vectors of the individuals that come from this population. Likewise, the other 25 populations can be represented by 25 points in the 12-dimensional space for each chromosome, and further analysis on genetic relationships among population are based on the average natural vectors for each chromosome.

The idea of taking an average plays a crucial role in our project, since it solves the problem of different sequences leading to different phylogenetic analyses. For alignment approaches, taking an ‘average’ alignment is not realizable, therefore further phylogenetic analysis does not lead to a convincing conclusion. However, alignment-free approaches are able to address this limitation by introducing mathematical concepts to the biological world, which is also one of the most common techniques in engineering. Averaging is both intuitively clear, and it is something easy to compute in practice.

We have also proposed an extension of the natural vector method, named Divided Natural Vector (DNV), based primarily on the idea of combining the natural vector method together with alignment algorithms. Alignment methods aim at finding the homologous positions among sequences. In contrast with the natural vector method, the alignment results are determined by local correspondence, rather than the global statistical information. The divided natural vector approach evaluates a sequence in two steps: first, we divide the sequence into k segments, where k is a positive integer; second, the 12-dimensional traditional natural vectors for each segment are concatenated into a single vector, with length of 12*k. When k = 1, the DNV approach degenerates to the case of traditional natural vector method. The computational complexity increases as k becomes larger, since more segments provide more direct evidence of local information than the rough statistics. The results of DNV could be the same as alignment approaches when an appropriate value of k is chosen.

In this project, we set k = 4 based on the testing of DNV, NV and MUSCLE on a published dataset (Briggs et al., 2009). The dataset including 55 samples was tested to perform the analysis, including the whole genomes of 46 modern individuals, one reference human genome, 6 Neanderthals and 2 other primates. This is a subset of the dataset used in (Briggs et al., 2009). The validation criterion was that the good approach should distinguish between the following groups: human and other primates, Neanderthals and modern individuals, 15 modern African and 31 non-African individuals. The UPGMA trees for the natural vector method, the divided natural vector (k = 4) method and MUSCLE are shown in Supplementary Figures S2A–C, respectively. All three approaches can distinguish between human and other primates, except the natural vector method fails to distinguish between Africans and non-Africans in Supplementary Figure S2A. Both the divided natural vector (k = 4) method and MUSCLE get satisfactory results, but MUSCLE takes about 1 hour 18 minutes, while DNV takes only seconds. Therefore, we have proved that the divided natural vector method extracts the information hidden in the original sequences more accurately than the traditional Natural Vector method, and is much more time-efficient compared to the alignment approach.

The DNV approach was also conducted on the same reconstructed sequences of all chromosomes and the results indicate that it does capture more information than the traditional natural vector method and the results are more stable.

### Euclidean Distance and the Phylogenetic Analysis

The workflow of this project is summarized in Supplementary Figure S3. After the original reconstructed sequences are converted to natural vectors (which correspond to points in real Euclidean space), the Euclidean distance between the points can be easily calculated to measure the difference between the sequences. For a specific chromosome, an average natural vector of each population is represented by the center of average personal natural vectors of chromosome pairs from all individuals who belong to this population. Thus, the distance between two populations can be depicted by the distance between two centers, i.e., two average natural vectors.

After the pairwise-distance matrix between populations was obtained, phylogenetic analysis was constructed in several ways in our project. FASTME provides distance algorithms to infer phylogenies based on balanced minimum evolution, which is the principle behind Neighbor-Joining (NJ). FASTME is an improvement over NJ because it performs topological moves using fast, sophisticated algorithms (Saitou and Nei 1987; Gascuel et al., 1997; Desper and Gascuel 2002; Desper and Gascuel 2004). We applied FASTME to the dataset analyzed with default parameters, and BIONJ, an improved version of the Neighbor-Joining algorithm based on a simple model of sequence data. In addition, the Unweighted Pair Group Method with Arithmetic mean (UPGMA) and Neighbor-Joining (NJ) are also straightforward ways installed in MEGA X (Sneath and Sokal 1973; Saitou and Nei 1987; Kumar et al., 2018) to construct phylogenetic trees and study the evolutionary relationships based on the distance matrix of all units involved. These three algorithms were all tested to construct reliable phylogenetic trees in our project.

Any distance matrix can be the input to these tree construction algorithms, as long as it is symmetric, diagonal elements are zero, and the remaining elements are positive. In order to present a comprehensive phylogenetic tree, all the 22 distance matrices derived from single autosome are summed together to obtain a Total Autosome Nucleotide Tree (TANT), containing information from all autosomes. The sum here is also an application of the idea of taking an average, since the sum and average only differs in one constant, and this substantial equivalence will lead to same results.

Another novel method to visualize the relationships between units is Natural Graph (Zheng et al., 2015). By finding the closest neighbor of all units in several layers (in most cases, two layers are enough), it successfully classifies units into several groups and also reveals the relationships between groups. Evidently, different chromosomes contribute to different average natural vectors, which leads to different phylogenetic results, and this principle applies to both phylogenetic trees and Natural Graphs.

### Filtration of Data From HmtDB and L0 Mitogenomes

We downloaded all mitochondrial sequences of each African country from HmtDB (https://www.hmtdb.uniba.it/query). Only complete genomes from healthy individuals on African continent are selected in this step. Since the length of human reference mitochondrial sequence is 16,569 bp, we deleted the sequences that are too short (<16,564 bp) or too long (>16,574 bp), and also the sequences that contain more than 1 “N”. Besides, each country contains at least two sequences that satisfy the criteria above. After the filtration, we obtained 2932 sequences from 30 African countries which can be used for evolutionary analysis.

For the L0 mitogenomes, we used the same criteria to select the reliable sequences, based on the length and the number of “N” in each sequence. Because the trees were constructed on the individual level for L0 mitogenomes, no country information is required in the phylogenetic analysis.

## Data Availability

The original contributions presented in the study are included in the Supplementary Material, further inquiries can be directed to the corresponding author.
