# Supplementary material for: Full Chromosomal Relationships Between Populations and the Origin of Humans
Source: Front Genet. 2022 Feb 2;12:828805. doi: 10.3389/fgene.2021.828805 (PMC8847220; doi:10.3389/fgene.2021.828805)
Supplement: Supplementary file 1 [file Presentation1.pdf]

Supplementary Material

1 Supplementary Data

1.1 Phylogenetic trees

1.1.1 Total autosome nucleotide tree (TANT)

Individuals in the 1000 Genomes Project are sampled from 26 populations, which are the representatives of 5 continental groups, i.e., superpopulations: Africa (AFR), Americas (AMR), East Asia (EAS), Europe (EUR) and South Asia (SAS). Both NV and DNV were tested on the dataset, with two ways to construct the evolutionary trees, UPGMA and NJ.

As shown in Extended Data Figure 4, all superpopulations except the Americas are divided clearly in both UPGMA and NJ trees, and the deletion of American populations in the whole dataset has greatly improved the stability of the classification. According to the 2013-2017 American Community Survey (U.S. Census Bureau, online survey), only 0.8% of the total population identify themselves as 'American Indian or Alaska Native' as their only origin, which indicates that the six American populations are actually a combination of humans from worldwide, rather than a pure human race in the evolutionary sense. It is therefore important to eliminate the noise that American populations may bring into the dataset. Please note that two populations (American Caribbean in Barbados (ACB) and African Ancestry in Southwest US (ASW)) whose origin are actually from Africa, are classified as American populations in 1000 Genomes Project, but their ancestors are both from Africa, and are clustered with African branch in all tested trees, hence they are assigned with the same color as African populations in Extended Data Figure 4 and 5.

In all the TANTs, African populations comprises a unique branch from the other non-African populations. This is consistent with many published literatures (Ingman et al. 2000; The 1000 Genomes Project Consortium 2012). Distances between populations within each continental group are calculated to measure the diversity of each superpopulations, as shown in Extended Data Table 1. The largest value is the distance between the six American populations (denoted as 'American1' in the table), and even after deleting ACB and ASW, the value (denoted as 'American2') is still larger than others. This demonstrate that American individuals are highly diverse and have great variability in populations, therefore they are not an ideal source to study human origin. For the other continents, populations of African ancestry harbor the greatest number of differences between populations on the genome-level, as predicted by the out-of-Africa model of human origins (The 1000 Genomes Project Consortium 2015).

1.1.2 Results based on single autosomes

Based on each pair of autosomes, both the BIONJ and UPGMA trees are also constructed as well, with or without American populations considered. The phylogenetic trees based on BIONJ and UPGMA shows limited differences, and in all trees, African populations and the two African-ancestry American populations together form a unique branch from others, and this is convincing for the out-of-Africa model again.

A significant improvement is observed when comparing the trees obtained by NV and DNV approaches, with or without noises in the dataset. When noise from American populations is included in the dataset, the topology of the trees is disturbed and it may even affect the classification in other continental groups. DNV is more robust in terms of removing these disturbances. For the traditional NV method, only trees based on data of Chromosome 2,3,6,12 and 15 are able to identify four nodes for African, East Asia, Europe and South Asia. When applying DNV to the same datasets, one can not only distinguish the four nodes on all chromosomes, but also infer the relationships between American populations and others as well. American populations barely cluster together, which proves again that the American populations are not from a single origin. An example is shown in Extended Data Figure 6(a) and Extended Data Figure 6(b), which represents the NV and DNV results for Chromosome 20, respectively. In Extended Data Figure 6(a), EUR_TSI (Toscani in Italy) is in the same branch as South Asia, rather than with other European populations; while in Extended Data Figure 6(b), this problem gets fixed.

After deleting the American populations in the dataset, NV fails to find four nodes for all autosomes, but DNV still works on most chromosomes. Extended Data Figure 6(c) and 6(d) gives an example of Chromosome 18, where again, for the result of NV, Toscani is not in the position where it is supposed to be but for the tree of DNV, is clustered together with EUR_IBS (Iberian population in Spain), same result as in Extended Data Figure 6(b), with noises included in the dataset.

Consistent with TANTs, most phylogenetic trees prove that non-American populations are monophyletic, and European and South Asian populations are more genetically related to each other than to East Asian populations. This inheritance pattern of autosomes is parental and an average of paternal and matrilineal inheritance, and the closer relationship between European and South Asian populations matches with the results based on chromosomes that are parental.

1.1.3 Sex chromosomes and mitochondrial genomes

All females in the 1000 Genomes Project have two X chromosomes, inherited from each parent respectively. The genetic mechanism of X chromosome should be very similar to that of autosomes. The only differences that occur are because all carriers are females. There is a significant improvement after altering the method from NV to DNV, and the BIONJ tree for DNV is shown in Extended Data Figure 7(a). The four superpopulations from the different continents are distinguished by different colors, and the Robinson-Foulds (Robinson and Foulds 1981) distances between the BIONJ trees of the X chromosome and each autosome are calculated as well in Extended Data Table 2. The R-F topological distance between two or more additive trees are commonly used in bioinformatics to determine tree similarity. This distance is equal to the minimum number of elementary operations, consisting of merging or splitting nodes, necessary to transform one tree into the other. The R-F distance between the X chromosome and Chromosome 18 is evidently smaller than others. As evidence of this, it has been observed that the Edward syndrome, also known as trisomy 18, a very frequent condition due to a third chromosome at birth, is more prevalent in female than male offspring. Though much more analysis is required to establish the relationship between gender and Trisomy 18, we believe that this might be interesting evidence.

In females, the X chromosome follows parental inheritance like the autosomes follow parental inheritance for both sexes. As shown in the results for autosomes (both TANTs and single autosome examples), European (blue) and South Asian (purple) populations have more similarity between each other, compared to East Asian (green) populations. The results in Extended Data Figure 7(b) show

different results. For this figure shows that European populations are in some ways closer to East Asian populations. This may be, due to its paternal inheritance pattern, in contrast with parental inheritance of autosomes and the X chromosome for females.

Besides, Extended Data Figure 7(b) shows a strange evolutionary pattern for males in the Finnish population. Finland is a country in Northern Europe bordering Russia to the east. Historical reasons may explain why it is closer to East Asia for males. Finland was incorporated into the Russian Empire in 1809, and remained part of Russia for over 1000 years until its declaration of independence in 1917. After that, its low fertility rate may have caused the Y chromosome with Asian ancestry to be conserved in the population. Russian, China and Thailand are of the largest groups that contribute to the Finnish populations. Another explanation for this is about the DYS7C deletion, a recurrent deletion on the long arm of the Y chromosome in normal males, which is confined to Asia, Australasia, and southern and northern Europe. For those with reasonable sample size (Jobling et al. 1996), Finnish had the highest deletion frequency.

The genetic pattern of males is revealed from the Y chromosome. For females, the genetic pattern is more related to mitochondrial genomes. An individual's mitochondrial genome is not inherited by the same mechanism as nuclear genomes, and usually comes from the egg only. Mitochondria are, therefore, in most cases inherited only from mothers, a pattern known as maternal inheritance. The BIONJ tree based on the dataset of mtDNA is shown in Extended Data Figure 8, and SAS_PJL, a population in South Asia, Punjabi in Lahore, Pakistan, is in the branch of East Asia. Lahore is the capital of the Pakistani province of Punjab and one of Pakistan's wealthiest cities, which may result in robust trade with other Asian countries. Please note that based on the female data, the X chromosome also indicates a closer relationship between Punjabi and East Asia populations in Extended Data Figure 7(a). Pakistan is bordered by China in the northeast, and to some extent, this explains why that it is closer to the EAS_CHS, Southern Han Chinese in China.

The relationships between non-African superpopulations change again based on the results of mtDNA dataset. East Asian and South Asian are closer in Extended Data Figure 8. Combined with previous results, maternal inheritance may explain this finding. Therefore, we have found that different inheritance patterns result in different phylogenetic relationships, and parental inheritance can be viewed as the average of paternal and matrilineal inheritance. However, it is consistent in all trees that the first major separation in the evolutionary tree of modern humans was between Africans and non-Africans.

We have confronted the computational challenges as well, but they were solved within a reasonable and acceptable computation times. All the computations were performed in parallel on a local server. We used CentOS 7 Linux Server running on a Dell PowerEdge R740 with Dual Intel Xeon Gold 6128 6C/12T CPU @3.40GHz and 384 GB RAM. Extended Data Table 3 presents the time required to reconstruct one sequence and to calculate its natural vector and divided natural vector, respectively. The reconstructed chromosomes are stored on our server and will be made public for other researchers' further analysis based on genomic data. We also plan to improve the reconstructed sequences by using higher coverage sequencing and more advanced techniques in the future, and with more accurate sequencing results. When we do this, the corresponding phylogenetic trees will better reflect the real evolutionary and relationships between populations.

1.2 Mitogenomes

In the dataset covering 3495 individuals from southern Africa and eastern Africa, we found that the closest individual to the root of the tree is from Ethiopia, which also suggests the eastern Africa as the origin of human.

The tree files can be found on GitHub (https://github.com/YaulabTsinghua/Human-Origin-1kGP).

2 Supplementary figures and tables

2.1 Supplementary figures

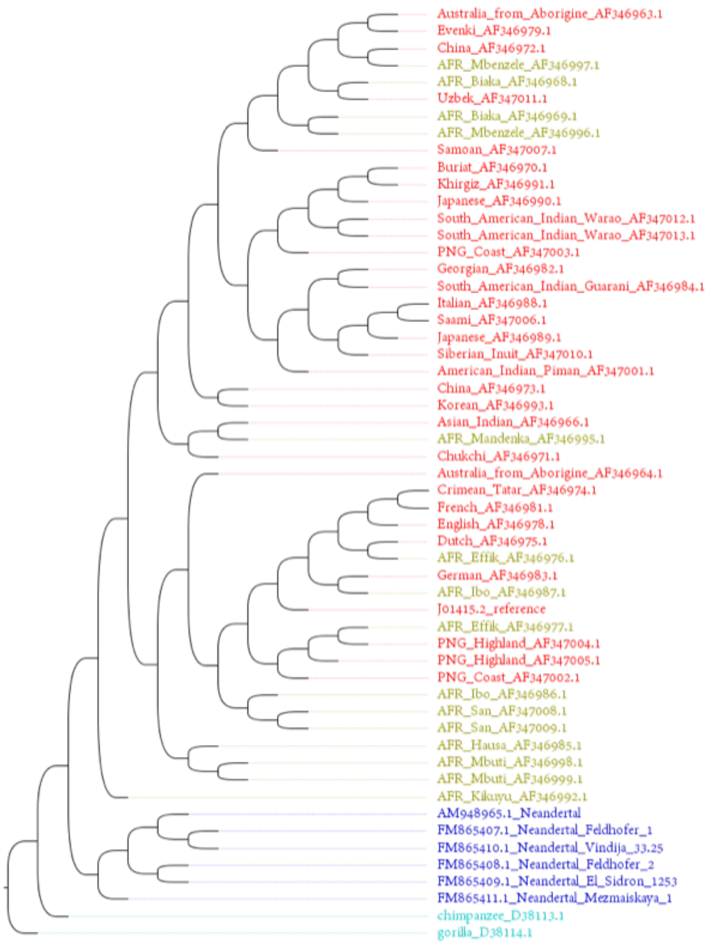

(a) UPGMA tree of the test set using Natural Vector method

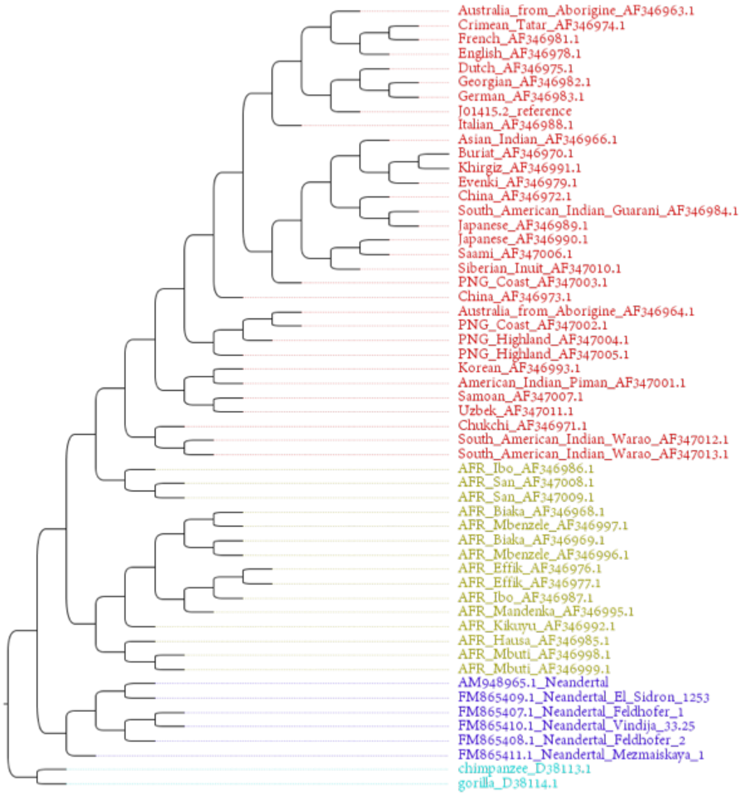

(b) UPGMA tree of the test set using Divided Natural Vector (k=4) method

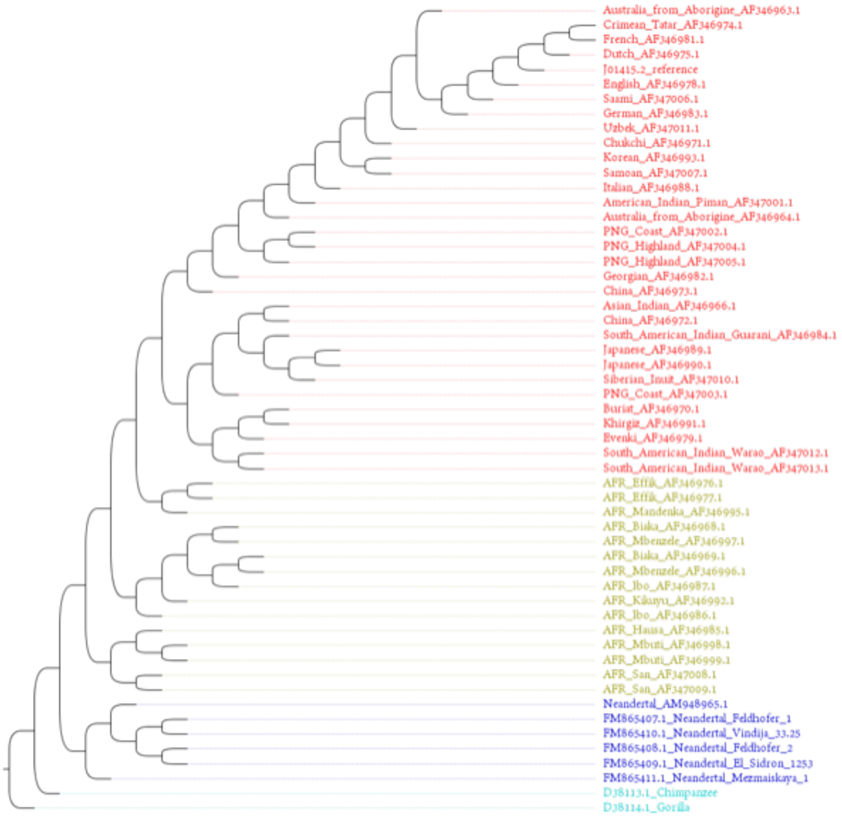

(c) UPGMA tree of the test set using MUSCLE

Supplementary Figure S1. The UPGMA trees of the 55 samples based on the (a) Natural Vector method; (b) Divided Natural Vector method (k=4); (c) MUSCLE.

#CHROM	POS	ID	REF	ALT	QUAL	FILTER	INFO	FORMAT	HG00096	HG01770	HG01992	HG02230	HG02231
MT	10	.	T	C	100	fa	VT=S;AC=3	GT	0	1	0	0	0
MT	16	.	A	T	100	fa	VT=S;AC=3	GT	0	0	0	0	0
MT	26	.	C	T	100	fa	VT=S;AC=3	GT	0	1	0	0	0
MT	35	.	G	A	100	fa	VT=S;AC=2	GT	0	0	0	0	0
MT	40	.	TC	CT	100	fa	VT=M;AC=1	GT	0	0	0	0	0
MT	41	.	C	T	100	fa	VT=S;AC=4	GT	0	0	0	0	0
MT	42	.	TCC	CCC,T	100	fa	VT=S,I;AC=1,1	GT	0	0	0	0	0
MT	46	.	T	C	100	fa	VT=S;AC=1	GT	0	0	0	0	0
MT	47	.	G	A	100	fa	VT=S;AC=1	GT	0	0	0	0	0
MT	52	.	TGG	CAA	100	fa	VT=M;AC=1	GT	0	0	0	0	0
MT	55	.	TATTTT	T,CATTTT,AATTTT,TTT,TTTTT	100	fa	VT=I,S,S,I,I;AC=5,3,2,1,1	GT	0	0	2	2	0

(a)

**Position**	1	2	3	4	5	6	7	8	9	10	11	12	13	14	15	16	17	18	19	20	21	22	23	24	25	26	27	28	29	30	…	51	52	53	54	55	56	57	58	59	60
**Reference**	G	A	T	C	A	C	A	G	G	T	C	T	A	T	C	A	C	C	C	T	A	T	T	A	A	C	C	A	C	T	…	T	T	G	G	T	A	T	T	T	T
**HG00096**	G	A	T	C	A	C	A	G	G	T	C	T	A	T	C	A	C	C	C	T	A	T	T	A	A	C	C	A	C	T	…	T	T	G	G	T	A	T	T	T	T
**HG01770**	G	A	T	C	A	C	A	G	G	C	C	T	A	T	C	A	C	C	C	T	A	T	T	A	A	T	C	A	C	T	…	T	T	G	G	T	A	T	T	T	T
**HG01992**	G	A	T	C	A	C	A	G	G	T	C	T	A	T	C	A	C	C	C	T	A	T	T	A	A	C	C	A	C	T	…	T	T	G	G	C	A	T	T	T	T
**HG02230**	G	A	T	C	A	C	A	G	G	T	C	T	A	T	C	A	C	C	C	T	A	T	T	A	A	C	C	A	C	T	…	T	T	G	G	C	A	T	T	T	T
**HG02231**	G	A	T	C	A	C	A	G	G	T	C	T	A	T	C	A	C	C	C	T	A	T	T	A	A	C	C	A	C	T	…	T	T	G	G	T	A	T	T	T	T

(b)

Supplementary Figure S2. An example of a VCF file in the 1000 Genomes Project

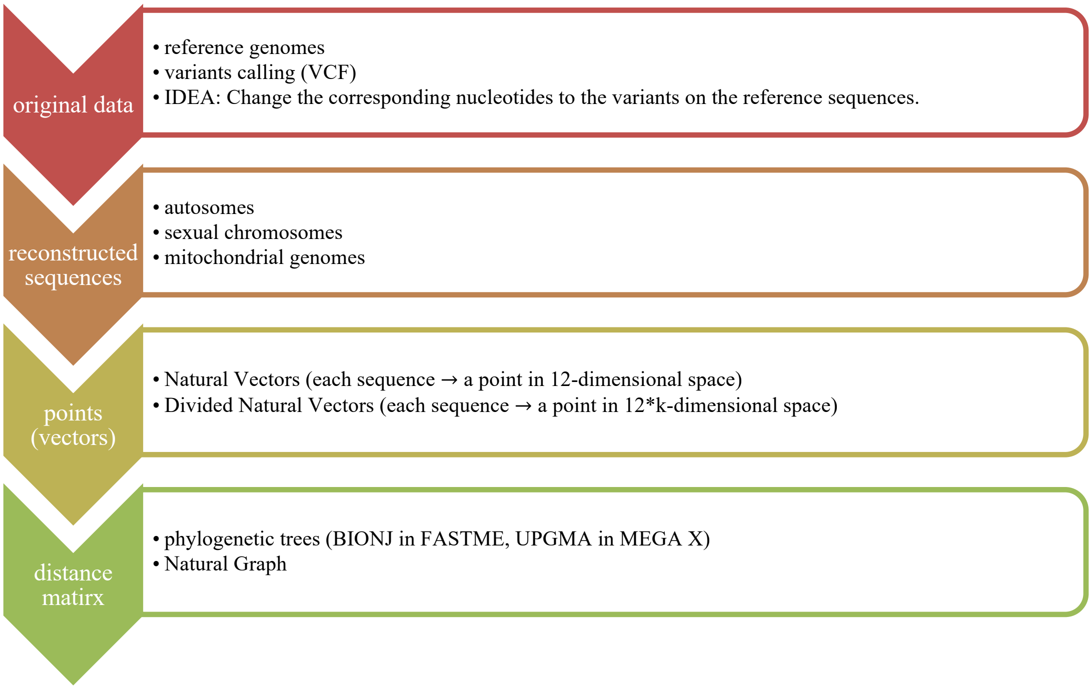

Supplementary Figure S3. Workflow of our analysis of relationships among human populations

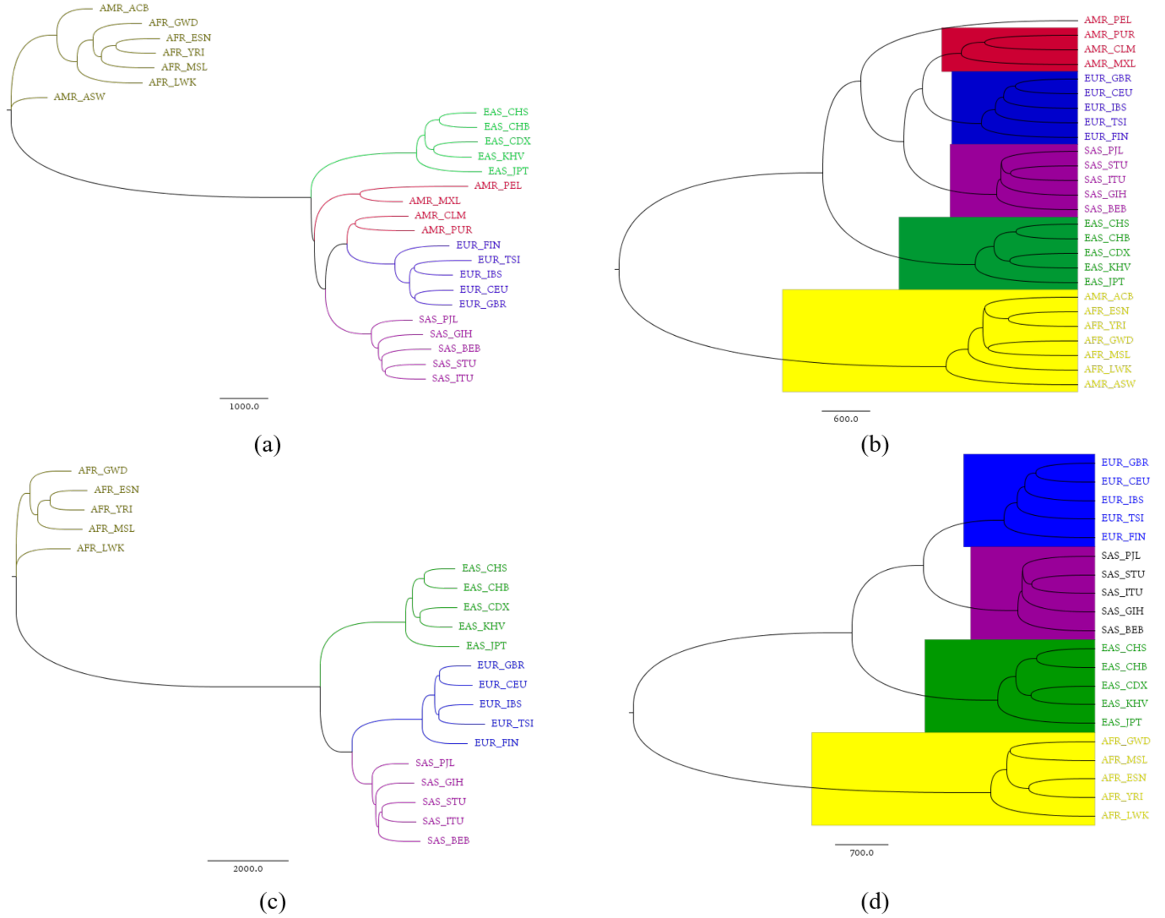

Supplementary Figure S4. The phylogenetic trees based on the Divided Natural Vector method, with k=4. (a) the Neighbor-Joining tree for all 26 populations, (b) the UPGMA tree for all 26 populations, (c) the Neighbor-Joining tree for 20 non-American populations and (d) the UPGMA tree for 20 non-American populations

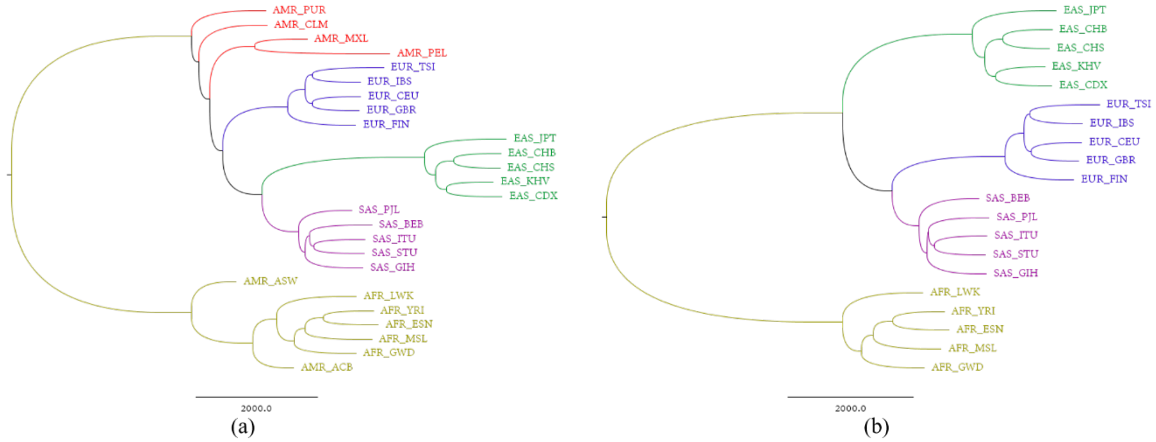

Supplementary Figure S5. The BIONJ TANTs based on two alignment-free methods based on the distance matrix obtained from (a) Divided Natural Vectors for all 26 populations (b) Divided Natural Vectors for 20 non-American populations

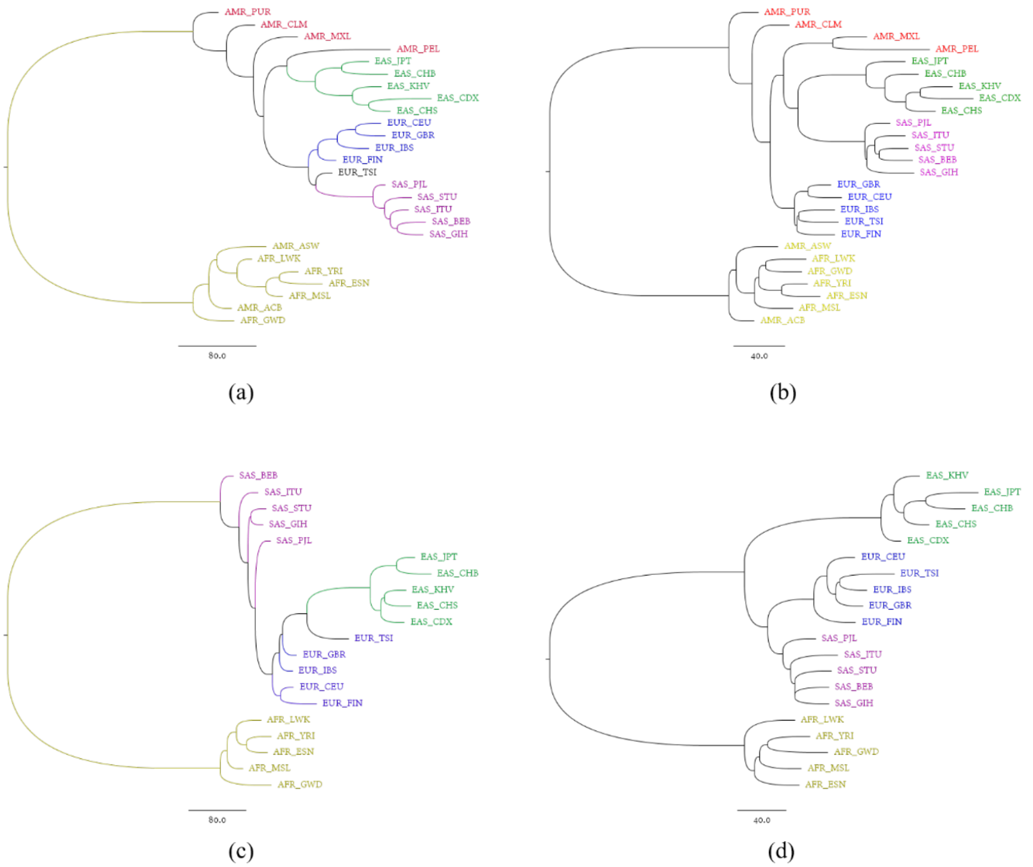

Supplementary Figure S6. The BIONJ trees based on two alignment-free methods based on the data of Chromosome 20 the distance matrix obtained from (a) Natural Vectors for all 26 populations, (b) Divided Natural Vectors for all 26 populations; based on the data of Chromosome 18 the distance matrix obtained from (c) Natural Vectors for 20 non-American populations and (d) Divided Natural Vectors for 20 non-American populations

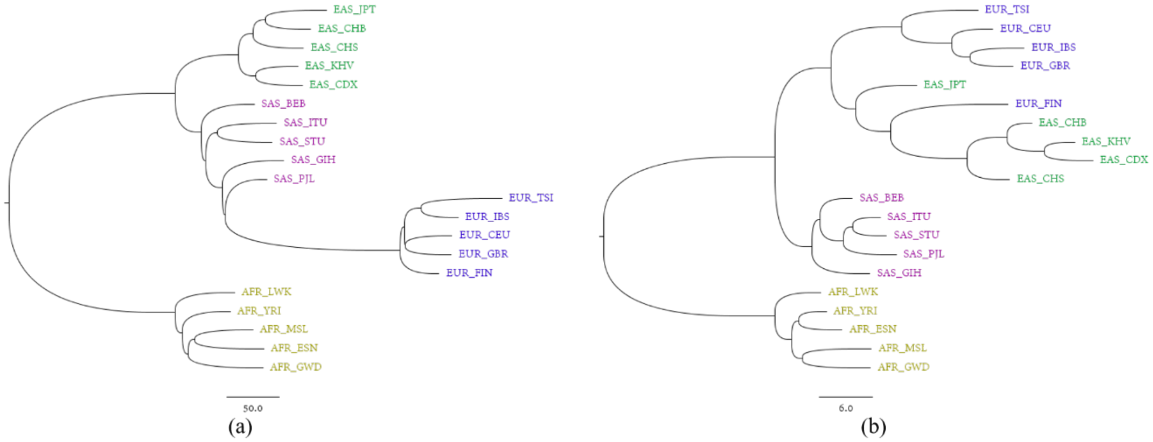

Supplementary Figure S7. The BIONJ trees based on the distance matrix obtained from DNV method based on the data of (a) Chromosome X, (b) Chromosome Y of 20 populations.

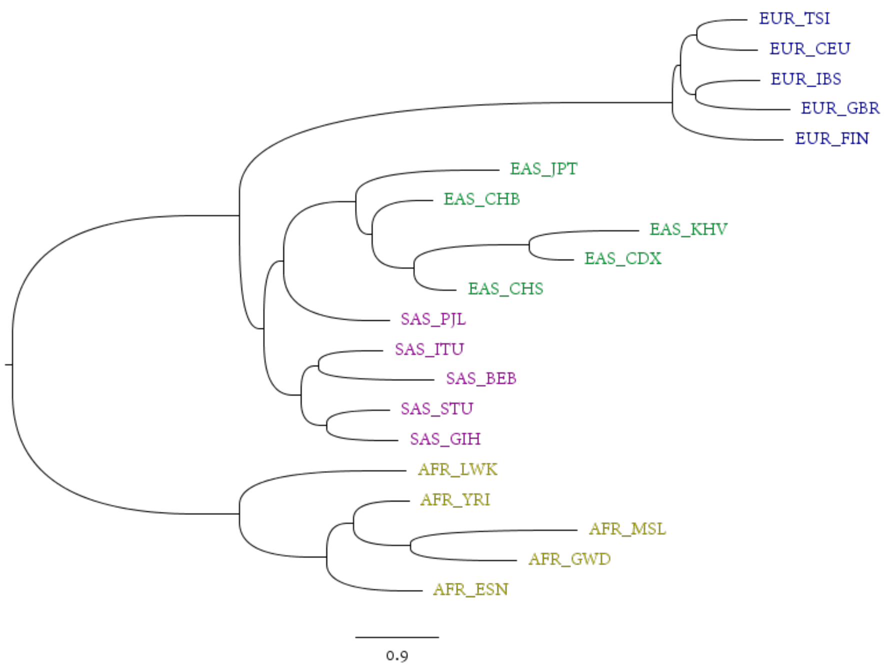

Supplementary Figure S8. The BIONJ trees based on the distance matrix obtained from DNV from the mitochondrial data of 20 populations.

2.2 Supplementary tables

**Autosome**	**Chr1**	**Chr2**	**Chr3**	**Chr4**	**Chr5**	**Chr6**	**Chr7**	**Chr8**	**Chr9**	**Chr10**	**Chr11**
**R-F distance**	18	16	20	24	16	24	12	20	20	22	22
**Autosome**	**Chr12**	**Chr13**	**Chr14**	**Chr15**	**Chr16**	**Chr17**	**Chr18**	**Chr19**	**Chr20**	**Chr21**	**Chr22**
**R-F distance**	20	18	16	20	18	24	8	24	20	18	16

Supplementary Table S1. The Robinson-Foulds distance between BIONJ trees based on Chromosome X and on autosome

**Superpopulation**	African	American1	American2	Europe	East Asia	South Asia
**Within Distance**	2817.54	9697.39	3984.00	2481.32	2600.40	2275.02

Supplementary Table S2. The distance within each superpopulation based on the data of all autosomes and NV approach

**Chr**	**1**	**2**	**3**	**4**	**5**	**6**	**7**	**8**	**9**
**Reconstruct sequence**	24m37s	27m29s	20m41s	20m27s	18m8s	17m46s	15m55s	14m7s	10m56s
**Calculate NV**	3m42s	3m14s	2m40s	2m39s	2m1s	2m31s	2m18s	1m58s	1m51s
**Calculate DNV (k=4)**	3m24s	2m50s	2m25s	2m18s	2m9s	1m57s	2m6s	1m44s	1m55s

Chr	10	11	12	13	14	15	16	17	18
**Reconstruct sequence**	13m4s	13m5s	12m23s	8m38s	6m39s	6m18s	6m55s	6m15s	5m49s
**Calculate NV**	2m12s	1m58s	1m50s	1m48s	1m22s	1m44s	1m10s	1m2s	1m9s
**Calculate DNV (k=4)**	1m37s	1m41s	1m32s	1m31s	1m34s	1m37s	1m16s	1m04s	0m42s
**Chr**	**19**	**20**	**21**	**22**	**MT**	**X**	**Y**		
**Reconstruct sequence**	4m31s	4m5s	2m19s	2m16s	<1s	10m46s	2s		
**Calculate NV**	0m52s	0m47s	0m53s	1m	<1s	1m58s	1m30s		
**Calculate DNV (k=4)**	0m57s	0m45s	0m48s	0m53s	<1s	1m50s	1m23s		

Supplementary Table S3. The time required to reconstruct one sequence from different chromosomes to calculate its natural vector and divided natural vector

3 References

Ingman M., Kaessmann H., Paabo S., et al. Mitochondrial genome variation and the origin of modern humans. 2000. Letters to Nature, 408:708-713.
Jobling M.A., Samara V., Pandya A., et al. Recurrent duplication and deletion polymorphisms on the long arm of the Y chromosome in normal males. 1996. Human Molecular Genetics, 5(11):1767–1775.
Robinson D. F., and Foulds L. R. Comparison of phylogenetic trees. 1981. Mathematical Biosciences, 53(1-2):131–147.
The 1000 Genomes Project Consortium. An integrated map of genetic variation from 1,092 human genomes. 2012. Nature, 491:56-65.
The 1000 Genomes Project Consortium. A global reference for human genetic variation. 2015. Nature, 526:68-74.
U.S. Census Bureau. "ACS Demographic and Housing Estimates – 2011–2015". Available from https://factfinder.census.gov/faces/tableservices/jsf/pages/productview.xhtml?src=bkmk
